# *ALK* Fusion in an Adolescent with Acute Myeloid Leukemia: A Case Report and Review of the Literature

**DOI:** 10.3390/biomedicines11071842

**Published:** 2023-06-27

**Authors:** Meghan Shekar, Gabriela Llaurador Caraballo, Jyotinder N. Punia, Choladda V. Curry, Kevin E. Fisher, Michele S. Redell

**Affiliations:** 1 Department of Pediatrics, Baylor College of Medicine, Houston, TX 77030, USA; gallaura@texaschildrens.org (G.L.C.); mlredell@texaschildrens.org (M.S.R.); 2 Texas Children’s Hospital, Houston, TX 77030, USA; jnpunia@texaschildrens.org (J.N.P.); cvcurry@texaschildrens.org (C.V.C.); kefisher@texaschildrens.org (K.E.F.); 3 Department of Pathology, Baylor College of Medicine, Houston, TX 77030, USA

**Keywords:** acute myeloid leukemia, targeted therapy, cytogenetics, ALK

## Abstract

Activating mutations and fusions of the *ALK* oncogene have been identified as drivers in a number of malignancies. Crizotinib and subsequent ALK tyrosine kinase inhibitors have improved treatment outcomes for these patients. In this paper, we discuss the case of an adolescent patient with acute myeloid leukemia, who was identified to have an activating *ALK* fusion, which is a rare finding and has never been reported in cases of AML without monosomy 7. Crizotinib was added to this patient’s frontline therapy and was well tolerated. In cases of more common gene alterations, existing data supports the use of targeted agents as post-HSCT maintenance therapy; however, crizotinib was not able to be used post-HSCT for this patient due to the inability to obtain insurance coverage.

## 1. Introduction

The *ALK* oncogene encodes ALK, a membrane-bound receptor tyrosine kinase, which activates multiple downstream signaling pathways that are keys in cell proliferation and survival [1]. It was first identified in 1994 as a fusion partner in a t(2;5) chromosomal translocation in anaplastic large cell lymphoma, which is how it got its name (Anaplastic Lymphoma Kinase) [2]. Many other fusion partners and activating gene alterations (point mutations, deletions, and rearrangements) have since been identified as oncogenic drivers in a variety of malignancies, including non-small-cell lung cancer (NSCLC), inflammatory myofibroblastic tumor (IMT), neuroblastoma, and renal cancers [3,4,5]. In the past two decades, the landscape of treatment for these tumors has been changed by targeted therapy with small-molecule ALK tyrosine kinase inhibitors (TKI), of which several are currently FDA approved for use in adults, and one (crizotinib) for use in children [6,7].

More recently, oncogenic *ALK* fusions have been identified in a small subset of patients with acute myeloid leukemia (AML) and other myeloid-lineage hematologic malignancies (Table 1). Recent advances in genomic and transcriptomic technologies have allowed us to perform risk stratification and adjust treatment for patients with AML based on cytogenetic and molecular features, but certain alterations continue to confer poor prognoses despite treatment intensification. Patients with the monosomy 7 subtype AML, a subset with a poor prognosis, were recently retrospectively evaluated via RNA sequencing by Ries et al. (2020), and 14% of the patients were found to have cryptic *ALK* fusions [8,9]. *ALK* fusions have been described in adults with the monosomy 7 subtype of AML as well [10,11,12]. Importantly, cells transformed by *ALK* fusions demonstrate cytokine-independent proliferation and are sensitive in vitro to crizotinib [9].

In this paper, we describe the case of an 18-year-old female with AML, who was found to have a somatic *RANBP2::ALK* fusion. This is the first reported case of AML with this *ALK* fusion *without monosomy* 7. We describe her treatment and clinical course, including the use of crizotinib. Just as patients with *FLT3*-mutated AML have shown improved overall survival from the addition of targeted therapy (sorafenib and other kinase inhibitors), both with upfront chemotherapy and as post-HSCT maintenance therapy [14,15], we believe crizotinib used at these same timepoints will benefit these rare patients with *ALK*-fusion AML.

## 2. Case Presentation

**Figure 1:**
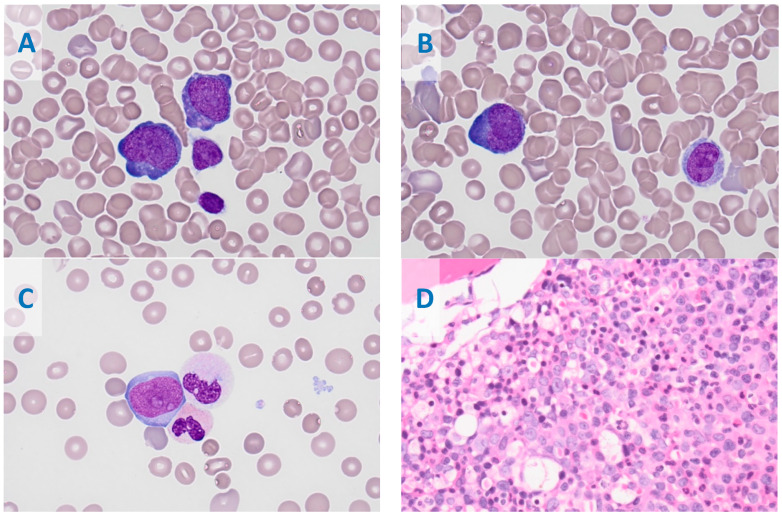
Bone marrow aspirate smears show increased leukemic blasts (20.5%). The blasts are medium to large sized with high N:C ratio, with round to irregular to slightly convoluted nuclear contours, and have a finely dispersed chromatin pattern and visible nucleoli with variably basophilic and rarely azurophilic granules; overall, the blasts have features of monoblasts (**A**) and promonocytes (blast equivalents (**B**). Occasional neutrophils are dysplastic with abnormal nuclear lobation and hypogranulation (**C**). The core biopsy shows leukemic blast infiltration (**D**); (**A**–**C**), Wright–Giemsa Stain, at 1000×; (**D**), Hematoxylin and Eosin stain, with a magnification of 400×.

**Figure 2:**
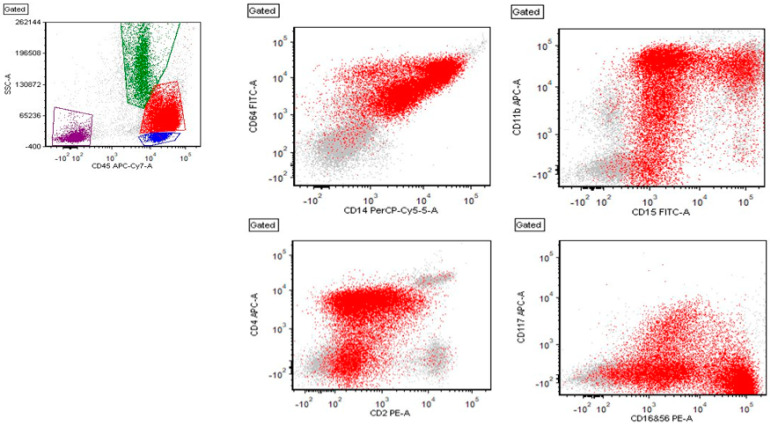
Flow cytometry showing red: leukemic blasts, green: granulocytes, blue: lymphocytes, and burgundy: debris. The blasts are positive for CD45, CD64, CD14, CD15, CD11b (partial), CD2 (partial), CD4, CD16+56 (partial, bright), and CD117 (partial) (shown here). The blasts are also positive for (not shown here) CD13, CD33, HLA-DR, CD52 (partial), CD99, CD58, CD38, and myeloperoxidase (partial).

An 18-year-old female presented to the emergency room at our hospital with one week of progressive fatigue and pallor. Her medical history included irregular menstrual periods and psychiatric diagnoses for which she took concomitant medications. She initially presented to a mobile clinic where a CBC was drawn, showing severe anemia. She was referred to the nearest pediatric emergency center, where a repeat CBC was significant, for a total WBC of 24.37 × 10^3^/µL with a monocytic predominance (45%) and no blasts present by automatic differential; hemoglobin of 5.8 g/dL; and platelet count of 30 × 10^3^/µL. An initial packed red blood cell (PRBC) transfusion was given with no significant increase in Hgb (post-transfusion Hgb of 6.0 g/dL). Her reticulocyte count, mean corpuscular volume (MCV), unconjugated bilirubin, and lactate dehydrogenase (LDH) were elevated, suggestive of possible acute hemolysis. The leading differential diagnosis at the time was acute leukemia, an autoimmune etiology causing destructive cytopenias, or a viral/infectious process. A flow cytometry analysis of peripheral blood identified a large population of monocytes, but without significant immunophenotypic aberrancies. Three days after admission, a bone marrow biopsy and aspirate were performed. The anatomic pathology revealed 20.5% blasts with features of abnormal monoblasts, which appeared more immature with prominent nuclear folds and gray to basophilic cytoplasm (Figure 1). The flow cytometry showed increased monocytes (46%) with immunophenotypic aberrancies, including CD4 (brighter), CD16+56 (partial, bright), CD117 (partial), and CD2 (partial), and a loss of CD11b (Figure 2). These results were consistent with acute myeloid leukemia with monocytic differentiation.

**Figure 3:**
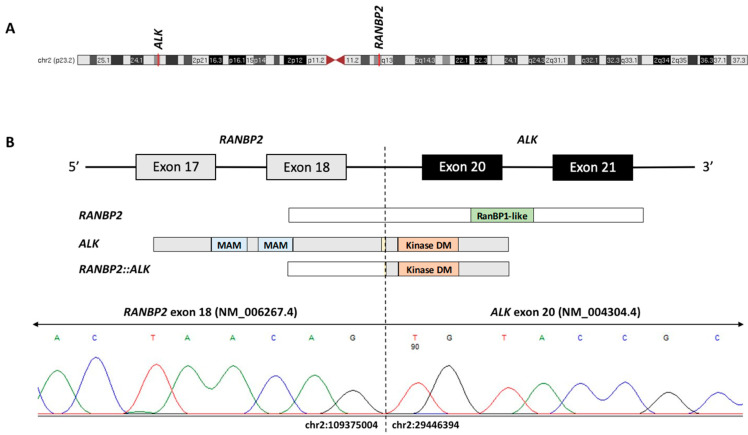
(**A**) Schematic of chromosome 2 with red lines denoting the chromosomal positions of *ALK* [ALK receptor tyrosine kinase] and *RANPB2* [RAN-binding protein 2] on chromosomes 2p23.1 and 2q13, respectively. (**B**) The *RANBP2*::*ALK* fusion protein is predicted to maintain an intact 3′ ALK tyrosine kinase domain (Kinase DM) by joining exon 18 of *RANBP2* to exon 20 of *ALK*. The RT-PCR and Sanger sequencing confirmed the fusion breakpoint at chr2:109375004 (*RANBP2*, NM_006267.4, exon 18) and chr2:29446394 (*ALK*, NM_004304.4, exon 20). Domain abbreviations: MAM, meprin/A5/mu; RanBP1-like, Ran-binding protein RanBP1-like.

The patient was transferred from the satellite community campus to the main academic campus and was enrolled into the active Children’s Oncology Group (COG) study for AML, AAML1831. She was randomized to Arm B with CPX-351 (Vyxeos; 60 mg/m^2^ daunorubicin equivalents IV on days 1, 3, and 5) and gemtuzumab ozogamicin (4.5 mg on day 6). A lumbar puncture on day 8 was consistent with the CNS1 status. On day 10 of therapy, the local cytogenetics studies with chromosome analysis showed an abnormal clone in 18 out of 20 cells characterized by a pericentric inversion of chromosome 2 at bands 2p23-q13. A fluorescence in situ hybridization (FISH) evaluation was performed using 13 probe sets for AML and MDS and revealed no evidence of abnormal signal patterns. At the same time, the results of her centralized molecular studies from the COG AAML1831 study showed no CEBPα or NPM1 mutation, and a *FLT3* internal tandem duplication (*FLT3*-ITD) with an allelic ratio of 0.62. Since the gilteritinib arm of the COG AAML1831 study was temporarily closed, she was taken off the protocol therapy the following day and started sorafenib at 400 mg PO daily on days 11–31. On day 27 of Induction 1, the patient’s primary oncologist was informed that the central lab’s *FLT3*-ITD result was an error due to sample mix-up. At the end of Induction 1 (day 45), an institutional next-generation DNA/RNA sequencing panel run on the diagnostic bone marrow aspirate was performed, confirming no *FLT3*-ITD mutation. The sequencing panel showed a fusion between exon 18 of RAN-binding protein 2 *(RANB2)* on chromosome 2 (2q13) and exon 20 of ALK receptor tyrosine kinase *(ALK)* on chromosome 2 (2p23.2-2p23.1). (Figure 3) This *RANBP2::ALK* fusion, which was presumed to retain the tyrosine kinase domain of *ALK*, was confirmed using RT-PCR and Sanger sequencing, and is consistent with the pericentric inversion of chromosome 2 noted in the initial diagnostic cytogenetic report.

A bone marrow evaluation at the end of Induction 1 showed a minimal residual disease (MRD) positive state with 0.1% residual acute myeloid leukemia via flow cytometry. Chromosome analysis was not able to be performed at this timepoint due to a markedly hypocellular (10%) marrow, resulting in inadequate sample volume being obtained. Based on this finding, the patient was considered high risk and therefore eligible for HSCT at first remission. She began Induction 2 and was treated according to an institutional practice standard with cytarabine (100 mg/m^2^/dose IV every 12 h on days 1–8) and daunorubicin (50 mg/m^2^/dose on days 1, 3, and 5). On day 10 of Induction 2, once systemic chemotherapy was completed, crizotinib at 250 mg twice daily was started as a targeted agent against the known oncogenic activating *ALK* fusion. A bone marrow evaluation at the end of Induction 2 demonstrated MRD-negative remission. She then received Intensification 1 as per the institutional practice standard of high-dose cytarabine (1000 mg/m^2^/dose every 12 h on days 1–5) and etoposide (150 mg/m^2^/dose on days 1–5), again with the addition of crizotinib beginning on day 7 of the cycle. The MRD evaluation remained negative after Intensification 1.

The patient tolerated the chemotherapy and crizotinib well. She experienced nausea that was managed with scheduled antiemetics; an erythematous rash of lower extremities that was likely secondary to CPX-351 and was managed with topical steroid and emollient; and episodes of febrile neutropenia during hospitalizations, but never with any bacterial or fungal organisms isolated. While on crizotinib, QTc was monitored closely and remained within the safe range to continue the medication.

Following three cycles of chemotherapy, she underwent an allogeneic hematopoietic stem cell transplant with a 10/10 HLA-matched unrelated donor. A conditioning regimen of alemtuzumab, busulfan, and cyclophosphamide was used. Her post-transplant course was complicated by severe shock with generalized fluid overload on day +12 in the setting of engraftment syndrome, as well as concomitant hepatic veno-occlusive disease/sinusoidal obstruction syndrome. As a result, she developed acute kidney injury that initially required continuous renal replacement therapy and then intermittent hemodialysis (last session on day +29). She was treated with 21 days of defibrotide (days +12 to +32). She engrafted on day +16 post-transplant. A day +30 disease evaluation of both bone marrow and CSF was performed, showing no evidence of leukemia cells based on flow cytometry, no evidence of *ALK* gene rearrangement based on FISH probe, and 100% donor cells. She was discharged from the hospital on day +39. A day +106 evaluation of bone marrow again showed no evidence of leukemia, no *ALK* gene rearrangement, and 100% donor cells. The initial plan was to begin crizotinib as a maintenance therapy once all cell lines recovered, estimated to be around day +100; however, its coverage was denied by her private insurance despite multiple appeals. She developed mixed chimerism initially noted during peripheral blood surveillance on day +168. Serial monitoring of donor chimerism status via peripheral blood using cytogenetics for the sex chromosome and short tandem repeat (STR)-PCR showed stable mixed chimera with >95% donor cells and without requiring additional interventions. The FISH evaluation of peripheral blood showed no evidence of *ALK* gene rearrangement despite mixed chimerism.

At the most recent follow-up visit, the patient was day +197 post-transplant and remains in remission. She had intermittent neutropenia requiring granulocyte colony stimulating factor (GCSF) in the setting of a viral respiratory infection which has resolved. She has a recent fluctuating low-level Epstein Barr virus (EBV) viremia not requiring treatment at this time. Her kidney function has improved. She has had no evidence of graft versus host disease, is now off immune-suppressive agents, and remains transfusion independent with stable, low-level mixed chimerism.

## 3. Discussion

The discovery and implementation of targeted cancer-directed therapy has thrust the field of oncology forward and improved survival for patients with various malignancies. In the treatment of AML, this has been best demonstrated by treatment of patients with FMS-like tyrosine kinase 3 internal tandem duplication (*FLT3*-ITD), an alteration present in about 30% of adult AML patients and 15% of pediatric AML patients. *FLT3*-ITD confers an especially poor prognosis with a high likelihood of relapse even after allogeneic stem cell transplant. However, the addition of TKIs that target FLT3, such as sorafenib, midostaurin, or gilteritinib, into frontline treatment in combination with conventional chemotherapy has reduced relapse rates and improved survival [16,17]. With this success, which is thought to be related to improved disease control before transplant, clinical practice has shifted toward also including a FLT3-targeting TKI as post-transplant maintenance therapy with the goal of reducing post-transplant relapses. While there is not yet a clear best practice or guideline for utilizing TKIs as post-transplant maintenance therapy in patients with *FLT3*-ITD+AML, many studies have shown survival benefits with tolerable toxicity profiles and costs [18]. The current Children’s Oncology Group frontline AML study, AAML1831, includes TKI (gilteritinib) maintenance therapy for one year after HSCT for patients with *FLT3*-ITD+AML.

Using this rationale, we hypothesized that adding the ALK-targeting TKI crizotinib to frontline treatment *and* as post-transplant maintenance therapy would benefit our patient. During upfront therapy, crizotinib was well tolerated and potentially contributed to the patient achieving and maintaining MRD negativity prior to HSCT. Unfortunately, because of the rarity of *ALK* fusions in AML, there are no existing prospective trial data to support prophylactic post-HSCT maintenance therapy in a MRD negative state for AML with *ALK* fusions, which has led to roadblocks in achieving insurance coverage of the medication.

In summary, this case study draws attention to a rare but targetable oncogenic fusion in a patient with AML, and it is the first reported case of an *ALK* fusion in AML *without* monosomy 7. Most institutional AML FISH probe sets do not include *ALK*; thus, we must rely on DNA and RNA sequencing as a crucial diagnostic tool to identify these and other rare, potentially actionable gene alterations. Our case study also highlights the challenges of our third-party payor health care system, which have substantial impact on physicians’ ability to use relevant, albeit limited, data to offer targeted agents off-label to patients with rare conditions.

## Figures and Tables

**Table 1:** Characteristics of patients reported in the literature to have myeloid-lineage hematologic malignancies with *ALK* fusions. Abbreviations: MDS, myelodysplastic syndrome; JMML, juvenile myelomonocytic leukemia; WBC, white blood cells; BM, bone marrow; ISCN, International System for Human Cytogenomic Nomenclature; RT-PCR, reverse transcription polymerase chain reaction.

Literature Source	Patient Age and Disease	Peripheral Blood and Marrow Description	Flow Cytometry Markers	ISCN	RT-PCR
Maesako et al. [10]	75 y, AML	WBC: 143.6 × 10^3^/µL 38.6% monocytes; 13.6% immature granulocytes (including blasts) BM blasts: 32.1% BM morphology: > 90% cellularity; marked hyperplasia of myeloid-lineage cells and monocytes	Pos: CD34, CD13, CD33, CD116, CD123, CD11b, CD11c, CD45RA, and HLA-DR +/−: CD36 and CD117 Neg: CD7 and CD56	46,XX,inv(2)(p23q13)/,45,idem, −7/46,idem, −7, +mar[1].ish inv(2)(p23)(3’ALK+) (q13)(5’ALK+)	*RANBP2::ALK*
Manselle et al. [9]	1.901 y, AML	WBC: 66.2 × 10^3^/µL BM blasts: 68%		45,XY,inv(2)(p13q14), −7[15]/46,XY[5]	*RANBP2::ALK*
	1.51 y, AML	WBC: 175.7 × 10^3^/µL BM blasts: 71.4%		45,XY,−7[24]	*SPTBN1::ALK*
	9.044 y, AML	WBC: 104.1 × 10^3^/µL BM blasts: 33%		45,XX,inv(2)(p21q21) c,−7[20]	*SPTBN1::ALK*
	1.244 y, AML	WBC: 99.8 × 10^3^/µL BM blasts: 49%		45,XX, −7[20]	*SPTBN1::ALK*
Lim et al. [12]	31.4 y, AML	WBC: 55.6 × 10^3^/µL; 11% blasts, 20 × 10^3^/µL monocytes BM blasts: 21.5% BM morphology: dramatically increased cellularity; increased myeloblasts and granulocytic hyperplasia	Pos: CD13, CD33, CD15, CD65, and CD14 Neg: CD117, CD41, CD7, CD34, TdT, and all B and T cell antigens	45,XX,inv(2)(p23q13),–7[20]	*RANBP2::ALK*
Rottgers et al. [13]	8.0 y, MDS or JMML	WBC: 84 × 10^3^/µL; 10% blasts; 34.5 × 10^3^/µL monocytes BM morphology: dramatically increased cellularity; hyperplasia and dysplasia of granulopoiesis		45,XY,inv(2)(p23q13), −7/46,XY[5]	* RANBP2::ALK *
	16.3 y, AML	WBC: 85.3K; 40% blasts; 22.5 × 10^3^/µL monocytes BM morphology: slightly increased cellularity; trilineage dysplasia		45,XY,inv(2)(p23q13), −7[8]/46,XY,inv(2)(p23q13)[3]	* RANBP2::ALK *
	3.5 y, JMML	WBC: 88 × 10^3^/µL; 20% blasts; 27 × 10^3^/µL monocytes BM morphology: dramatically increased cellularity; granulocytic hyperplasia		45,XY,t(2;2)(p23;q11~ 13), −7	* RANBP2::ALK *

## Data Availability

No new data were created or analyzed in this study. Data sharing is not applicable to this article.
